# Methylation of the Vitamin D Receptor Gene in Human Disorders

**DOI:** 10.3390/ijms25010107

**Published:** 2023-12-20

**Authors:** Beatrice Gasperini, Angela Falvino, Eleonora Piccirilli, Umberto Tarantino, Annalisa Botta, Virginia Veronica Visconti

**Affiliations:** 1 Department of Biomedicine and Prevention, University of Rome “Tor Vergata”, Via Montpellier 1, 00133 Rome, Italy; beatrice.gasperini@alumni.uniroma2.eu (B.G.); angela.falvino@students.uniroma2.eu (A.F.); virginia.veronica.visconti@uniroma2.it (V.V.V.); 2 Department of Clinical Sciences and Translational Medicine, University of Rome “Tor Vergata”, Via Montpellier 1, 00133 Rome, Italy; eleonora.piccirilli@ptvonline.it (E.P.); umberto.tarantino@uniroma2.it (U.T.)

**Keywords:** vitamin D receptor (VDR), epigenetics, DNA methylation, VDR-related diseases

## Abstract

The Vitamin D Receptor (VDR) mediates the actions of 1,25-Dihydroxvitamin D3 (1,25(OH)_2_D_3_), which has important roles in bone homeostasis, growth/differentiation of cells, immune functions, and reduction of inflammation. Emerging evidences suggest that epigenetic modifications of the *VDR* gene, particularly DNA methylation, may contribute to the onset and progression of many human disorders. This review aims to summarize the available information on the role of *VDR* methylation signatures in different pathological contexts, including autoimmune diseases, infectious diseases, cancer, and others. The reversible nature of DNA methylation could enable the development of therapeutic strategies, offering new avenues for the management of these worldwide diseases.

## 1. Introduction

**Figure 1:**
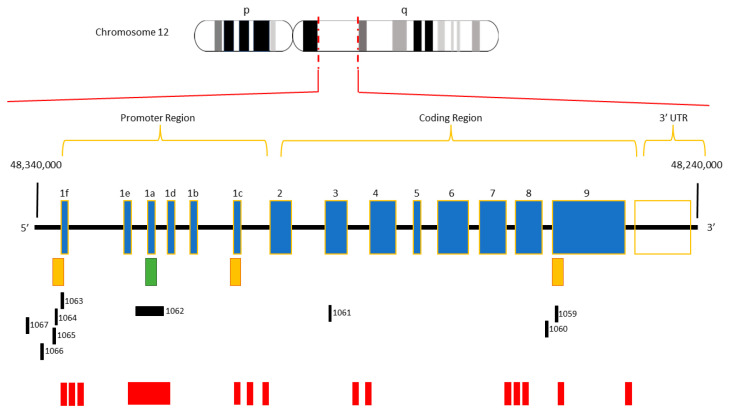
Structure of the human *VDR* gene. The human *VDR* is located on the long arm of chromosome 12 (12q12–q14) and comprises eight exons (2–9) along with six non-coding exons (1f–1c). The green box represents the primary promoter, and the orange boxes indicate the relative positions of *VDR* alternative promoters. The black boxes represent the *bona fide* CGIs mapped according to the CGIs predictive model by Bock and colleagues. The red boxes symbolize CGIs identified by bioinformatic tools.

The Vitamin D receptor (VDR) belongs to the nuclear receptor superfamily and can mediate the actions of the biologically active form of 1,25-Dihydroxvitamin D3 (1,25(OH)_2_D_3_) [1]. The binding of the ligand to the VDR induces a conformational change that modulates the interaction with several nuclear proteins. The VDR typically forms a heterodimer with the retinoid X receptor (RXR), which binds to genomic VDR binding sites within promoter regions [2,3,4,5]. This VDR-induced regulation of gene expression orchestrates key biological processes, including tumor progression, the immune system regulation, and cell proliferation and differentiation [6]. The *VDR* gene is located on the long arm of chromosome 12 (12q12-q14) and spans a genomic region of approximately 75 Kb. The gene comprises eight exons (2–9) that code for a protein of 427 amino acids, while six noncoding exons (1a–1f) are located in the 5′ regulatory region [7,8] (Figure 1). The human *VDR* primary promoter spans exon 1a and is characterized by the lack of a TATA initiator box, its GC-rich nature, and the presence of putative binding sites for a variety of transcription factors [7]. Three other alternative promoters are present in the *VDR* gene, located at exons 1f, 1c, and 9, some of which are tissue-specific [4] (Figure 1). Genetic variability, along with epigenetic modifications, can modulate *VDR* expression, contributing to the onset and progression of several diseases, generally known as “VDR-related” [4,9]. Among the epigenetic signatures, DNA methylation appears to be the most suitable biomarker for the detection and treatment of human diseases. DNA methylation is a mechanism that involves the transfer of a methyl group to the C5 position of cytosine, forming 5-methylcytosine, by enzymes belonging to the DNA methyltransferases (DNMTs), capable of transferring CH3 from S-adenyl Methionine (SAM) to the cytosine residue [10]. According to the predictive model of CpG islands (CGIs) made by Bock and colleagues [11], the *VDR* methylation can occur on 9 *bona fide* CGIs, which lie along the promoter regions and the gene body. In particular, two CGIs seem to play a crucial role in human diseases: CGI 1062 which overlaps exon 1a and comprises 56 CpG sites, and CGI 1060 overlapping with one of the alternative promoters (Figure 1) [11,12]. In addition, other GC-rich regions within the *VDR* gene have been identified using predictive methylation analytical tools, such as DataBase of CpG islands and Analytical Tool (DBCAT) (Figure 1) [13]. Given the complexity of the CpG content in the *VDR* gene sequence, the global characterization of the methylation pattern in different pathological conditions remains a major challenge to be fully explored. Indeed, dynamic *VDR* methylation signatures in specific CpGs may play a pivotal role in both the diagnosis and prognosis of several *VDR*-related diseases. These epigenetic changes can also vary in relation to environmental factors, such as light exposure and drug consumption [14,15]. The main purpose of this literature review is to provide an up-to-date overview of the role of *VDR* methylation in different human disorders, including autoimmune and infectious diseases, cancer, and others. A deeper understanding of disease-specific epigenetic patterns is critical to developing targeted and effective novel epigenetic therapies to improve the clinical management and responses to 1,25(OH)_2_D_3_.

## 2. Search Strategy

A global search of publications was conducted using the international bibliographic database PubMed. The identified articles until July 2023 were selected. The keywords used for the search were as follows: “VDR methylation”, “VDR methylation in diseases”, “VDR methylation in autoimmune diseases”, “VDR methylation AND Rheumatoid Arthritis”, “VDR methylation AND Multiple Sclerosis”, “VDR methylation AND Bechet’s disease”, “VDR methylation in infectious diseases”, “VDR methylation AND Tuberculosis disease”, “VDR methylation AND Hand Foot and Mouth disease”, “VDR methylation AND HIV”, “VDR methylation in cancer”, “VDR gene methylation AND breast cancer”, “VDR methylation AND Adrenocortical carcinoma”, “VDR methylation AND Hepatocellular carcinoma”, “VDR methylation AND Parathyroid adenomas”, “VDR methylation AND colorectal cancer”, “VDR methylation AND Osteoporosis”, “VDR methylation AND Male infertility”, “VDR methylation AND Recurrent Kidney stone formation”, and “VDR methylation AND Type 2 Diabetes Mellitus”. The search was conducted without restriction of ethnicity or geographic area, and language filters were applied to eliminate non-English language articles. In addition, the following exclusion criteria were used: abstracts, short communications, letters to the editor, and dissertations.

## 3. Autoimmune Diseases

**Figure 2:**
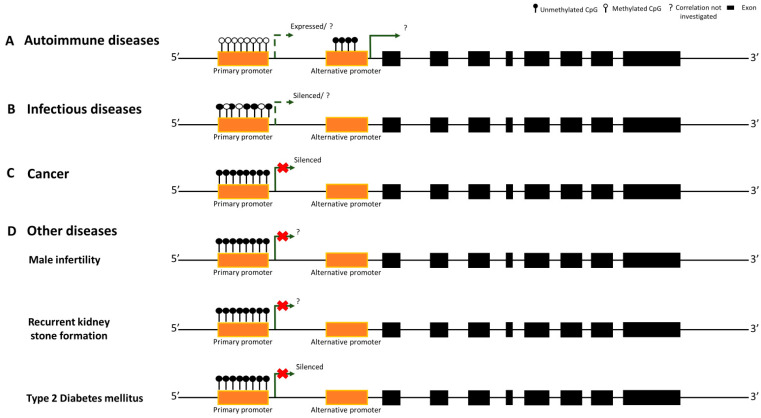
Schematic outline of the correlation between *VDR* methylation signature and gene expression pattern in autoimmune diseases (**A**), infectious diseases (**B**), cancer (**C**), and other diseases (**D**). Orange boxes represent the *VDR* promoter regions. The question mark indicates the lack of studies correlating the methylation status of the *VDR* gene and its expression level.

1,25(OH)_2_D_3_ modulates immune responses by regulating B and T-cell (TCs) proliferation and the production of inflammatory cytokines and immunoglobulin, including auto-antibodies [16]. 1,25(OH)_2_D_3_ plays a role in the activity of CD4+ TCs, enhancing the generation of regulatory T cells (Tregs) and Th2 cells and suppressing pro-inflammatory TCs, specifically Th17 and Th1 [16,17]. This regulatory mechanism is capable of creating a tolerogenic immune status which can be altered when the level of 1,25(OH)_2_D_3_ decreases, leading to immune dysregulation and increased susceptibility to autoimmune disorders [18,19]. The function of the 1,25(OH)_2_D_3_ is mediated by its binding with the VDR on the membrane of immune system cells such as dendritic cells, monocytes, and B and T lymphocytes [20]. Consequently, the genetic variability and epigenetic modifications in the *VDR* could alter immune homeostasis, significantly impacting the onset and progression of autoimmune diseases, such as rheumatoid arthritis (RA), multiple sclerosis (MS), and Behcet’s disease (BD) [21,22,23,24]. RA is a progressive autoimmune disease characterized by chronic joint inflammation and pain, attributed to the influx of CD4+ TCs into the synovial lining and the increase in macrophage-like and fibroblast-like cells that produce degradative enzymes and proinflammatory cytokines [25]. Although many studies have investigated the role of the *VDR*’s genetic variability in RA susceptibility, only a limited number of studies have been focused on the epigenetic signatures of this gene (Table 1). Zhang et al. investigated the methylation levels of the *VDR* primary promoter in a Chinese cohort comprising 122 RA patients and 123 controls (CTRs). The study identified a significant reduction in the *VDR* methylation levels in RA patients compared to CTRs, suggesting that this signature may represent a potential disease biomarker [26]. In contrast with these results, a second epigenetic study revealed no significant differences in the methylation levels of 10 CpG sites within the *VDR* primary promoter between a group of 35 RA patients and 41 healthy subjects [22]. However, this controversial result could be attributed to both the small size of the study cohort and the small number of CpG sites analyzed. 1,25(OH)_2_D_3_ and its receptor also play a pivotal role in multiple sclerosis (MS), as low levels of 1,25(OH)_2_D_3_ and the circulating form of 25-hydroxyvitamin D3 (25(OH)D_3_) or alterations in the *VDR* gene are a risk factor for this condition [27]. MS is a chronic and severe systemic autoimmune disease with abnormal immune-system reactions, which damage the central nervous system components [28]. The pathogenesis is still unclear, and recent data suggest the involvement of epigenetic mechanisms in MS susceptibility [29] (Table 1). So far, only one study has investigated the role of *VDR* methylation in T cells from relapsing-remitting MS (RRMS) patients compared to CTRs. The results revealed a significant increase in DNA methylation levels within an alternative promoter of the *VDR*, while the *VDR* primary promoter remains extensively unmethylated [23]. The specific pattern of *VDR* methylation in the alternative promoter at exon 1c suggests an important role of this region in MS susceptibility, which is expected to be further explored. One hypothesis suggested by the authors is the presence of a 4-exon transcript (GenBank accession: AK129594), which is a natural antisense transcript (NAT), that could modulate the *VDR* expression levels also through self-regulatory circuits, but this regulatory mechanism is still unclear [23]. Epigenetic changes of the *VDR* gene have also been investigated in Behcet’s disease (BD) (Table 1), a chronic recurrent multisystem inflammatory condition caused by several pro-inflammatory cytokines regulated by 1,25(OH)_2_D_3_ [24]. The study by Shirvani et al. analyzed the methylation levels of CpG sites in the *VDR* primary promoter (800 bp upstream and 200 bp downstream of the ATG start site) in peripheral blood mononuclear cells (PBMCs) from BD patients with respect to CTRs. Despite the decreased *VDR* expression levels observed in BD patients, the methylation level of the *VDR* gene reflected no differences between the two groups. This result may be explained by the effects of other GC-rich regions along the *VDR* gene not analyzed in this study or by additional epigenetic mechanisms leading to the observed *VDR* downregulation [30]. Overall, the study on the role of *VDR* methylation pattern in autoimmune diseases still remains a controversial and under-explored area in current research. The main reason may derive from the complexity of these disorders, whose underlying mechanisms have yet to be fully characterized (Figure 2). 

## 4. Infectious Diseases

1,25(OH)_2_D_3_ also plays a pivotal role in innate immune response, being able to stimulate immune cells to fight pathogens, such as *Mycobacterium tuberculosis*, enterovirus 71, and human immunodeficiency virus (HIV) [34,45,46]. The 1,25(OH)_2_D_3_-bound VDR induces the transcription of genes encoding antimicrobial peptides, such as cathelicidin and β-defensin 2, that contain *VDR* binding sites in their promoters. These peptides exhibit broad-spectrum antimicrobial activity against bacteria, viruses, and fungi, and their decrease has often been linked to low 1,25(OH)_2_D_3_ levels and increased susceptibility to infectious diseases [47,48,49]. A close interconnection exists between the progression of tuberculosis (TB) and deficiency of 1,25(OH)_2_D_3_ and 25(OH)D_3_ [50]. TB is caused by *Mycobacterium tuberculosis* infection, and its elimination depends on the expression of several antimicrobial peptides and cytokines, which are activated by the VDR [50,51]. A pilot study by Yang et al. identified an inverse correlation between the *VDR* hypermethylation pattern and the expression level of *VDR*, *IL-1β*, *IL-6*, and *TNF-α* genes in RAW 264.7 cells previously infected with *Mycobacterium*, suggesting a protective effect of the *VDR* hypomethylation signature against *Mycobacterium* infection [52]. A subsequent study performed by the same research group analyzed *VDR* methylation levels of a 269 bp region in the primary promoter comprising 16 CpG sites in TB patients compared to CTRs. The results showed an increase in *VDR* methylation levels in the TB patients vs. CTRs, confirming the potential involvement of this factor in the pathogenesis and progression of TB [31,52] (Table 1). Accordingly, another investigation carried out in children with active TB demonstrated an inverse correlation between *VDR* methylation and its expression levels. Most importantly, the hypermethylation of CpG sites in the *VDR* promoter region showed a significant diagnostic accuracy in discriminating TB disease, as determined by receiver operating characteristics (ROC) curve analysis [37] (Table 1). All these evidences support the hypothesis that increased *VDR* methylation causes cells of innate immunity to secrete insufficient antimicrobial peptides to fight TB disease [13]. Only one epidemiological study, conducted by Wang et al. in a Chinese population, does not support this model. The research analyzed 60 CpG sites within the *VDR* primary promoter in 122 TB patients vs. 118 CTRs, showing an opposite pattern of methylation with respect to the previous reports: *VDR* promoter hypomethylation in TB patients and *VDR* promoter hypermethylation in CTRs (Table 1). The discrepancy in the *VDR* methylation pattern observed in these three TB studies could have resulted from the different regions of the *VDR* analyzed. While the first two studies analyzed the *VDR* methylation in alternative promoter regions, Wang et al. focused their attention on the primary promoter. However, the latest study did not assess the downstream immune responses modulated by the binding of 1,25(OH)_2_D_3_ to the VDR and the impact of VDR methylation on its expression levels [32]. The methylation of *VDR* has also been implicated in the gravity of hand, foot, and mouth disease (HFMD) caused by enterovirus 71 (EV71) (Table 1) [33]. Li et al. evaluated the methylation status of the *VDR* primary promoter (from −638 bp to −545 bp of the ATG start site) in 58 children with severe HFMD, 58 children with mild HFMD, and 60 matched healthy controls. The results showed a hypermethylation of the *VDR* primary promoter in mild HFMD patients compared to children with severe HFMD, suggesting that *VDR* methylation is an early event in EV71-associated HFMD [33]. Among infectious diseases, recent data on the role of *VDR* epigenetic mechanisms in HIV disease are also emerging (Table 1). HIV is a retrovirus capable of infecting human cells, leading to an acquired immunodeficiency syndrome (AIDS) with a weakened immune system [53]. HIV seems to promote *VDR* methylation at its promoter region, inducing the downregulation of *VDR* expression levels in TCs [34]. A pilot study analyzed the methylation levels of CpG sites in the *VDR* primary promoter region in HIV-infected TCs, showing an increased methylation of CpG sites and increased expression of DNA methyltransferase 3b (*DNMT3b*) [34]. HIV-induced hypermethylation of the *VDR* in TCs led to a reduction in *VDR* expression levels, which promotes TCs apoptosis [34]. In conclusion, the majority of the studies reported here agree that *VDR* hypermethylation is a hallmark of increased susceptibility to infectious diseases (Figure 2). The identification of the complete the *VDR* epigenetic signature is, therefore, essential to understand the maintaining of a balanced immune homeostasis essential to efficiently counteract pathogens.

## 5. Cancer

The development and progression of cancer depend on multiple molecular mechanisms, in which 1,25(OH)_2_D_3_ and its receptor are highly involved [54]. In the pathological context of cancer, the VDR can directly or indirectly regulate the expression of >200 genes that influence cell proliferation, differentiation, and apoptosis, as well as immunomodulation and angiogenesis [55,56]. Several molecular pathways in cancer development are intimately linked to 1,25(OH)_2_D_3_. c-MYC, a protein that regulate the cell cycle often altered in cancer, contains VDR binding sites in its promoter [57]. It was reported that a ligand-activated *VDR* could downregulate c-MYC expression through a direct bond with two VDR binding sites [57,58]. Low levels of 1,25(OH)_2_D_3_ can also limit the hyperproliferative action of β-catenin in the epidermal cancer, interfering with the essential Wnt/β-catenin signaling [57,59]. Since genetic and epigenetic changes can influence the risk of developing cancer, many studies have been focused on the epigenetic signatures of the *VDR* gene [57]. Increased *VDR* methylation levels have been observed in several cancer types and lead to reduced mRNA and protein expression levels [35,60], supporting the loss of an antiproliferative role of the VDR [61]. *VDR* hypermethylation has been associated with hepatocellular carcinoma (HCC) [37], adrenocortical carcinoma (ACC) [36], colorectal cancer [39], and breast cancers [35] (Table 1)). Breast cancer is the most common cancer among women, affecting millions of people worldwide [62]. Its development is influenced by a complex interplay of genetic, epigenetic, and environmental factors [63]. 1,25(OH)_2_D_3_, by binding to its receptor, regulates the differentiation of the normal mammary gland and may be a useful parameter in treating or preventing breast cancer [35,64]. However, many breast cancer cells are resistant to 1,25(OH)_2_D_3_, but how this insensitivity may be involved in pathogenesis remains unclear [35]. Marik et al. analyzed the mechanism of resistance and the role of epigenetic silencing of the *VDR* by promoter hypermethylation [35]. An in silico analysis of the *VDR* gene identified three CGIs in an area ranging from −789 bp upstream to +380 bp downstream of the transcription start site (TSS) relative to the primary promoter [35]. The subsequent in vitro analysis conducted on different breast cancer and immortalized normal breast epithelial cell lines, previously treated with 1,25(OH)_2_D_3_, confirmed the insensitivity to calcitriol in breast cancer cells. Additionally, breast cancer cell lines showed an increase in *VDR* methylation levels than normal cells. To validate these preliminary data, the methylation analysis was also performed on breast cancer tissue compared to normal breast tissue. In breast cancers, the *VDR* promoter was significantly hypermethylated (65%) than in normal breast tissue (15%), supporting the proposed mechanism of silencing *VDR* expression through promoter hypermethylation, which appears linked to 1,25(OH)_2_D_3_ sensitivity [35]. Aberrant *VDR* promoter methylation has also been observed in human ACC, resulting in the dysregulation of steroid biosynthesis and adrenal growth [65,66]. A methylation analysis of a CGI located in the *VDR* primary promoter, including 42 CpG sites, was performed on ACC samples, revealing a higher methylation pattern of 27 CpG sites from 3/8 ACC samples. In addition, the hypermethylation of 27 *VDR* CpG sites has been shown to be correlated with a lower *VDR* mRNA expression [36]. Accordingly, another study showed that a percentage of *VDR* promoter methylation was significantly higher in the HCC group compared to both the chronic liver disease (CLD) and CTRs [37]. In contrast, Varshney et al. reported no promoter methylation of the *VDR* in parathyroid adenomas, although there was a 93% decrease in *VDR* expression in the same samples, highlighting the hypothesis that other mechanisms may regulate *VDR* levels [38]. Colorectal cancer is a third example in which the role of *VDR* promoter hypermethylation has been investigated. Colorectal carcinogenesis results from genetic and epigenetic changes as intestinal epithelial cells progress from average to malignant phenotypes [67]. A significant association was found between *VDR* methylation and *VDR* expression in colorectal cancer progression [39]. A study conducted by Afshan and colleagues in 75 colorectal cancer samples detected the hypermethylation of the *VDR* promoter in 28 (37.33%) of 75 cases [39]. Furthermore, methylation levels and decreased *VDR* expression were correlated with the different grades and stages of carcinoma progression, from poorly differentiated colorectal cancer to advanced stages [39]. Overall, studies conducted to date agree that *VDR* hypermethylation may favor cancer onset and progression (Figure 2). However, these analyses have been performed on a relatively small number of samples in a few cancer types. It is, therefore, necessary to fill these gaps before considering *VDR* methylation signature as a biomarker of different grades and stages of cancer progression.

## 6. Other Diseases

25(OH)D_3_ deficiency is a common background in many other human disorders, such as bone diseases, male infertility, type 2 diabetes mellitus (T2DM), and kidney stone formation [68,69,70,71]. The shared low 25(OH)D_3_ status prompted researchers to investigate the epigenetic signatures of the *VDR* in all these pathological conditions (Table 1). Recent studies have explored the DNA methylation pattern of several genes, including the *VDR*, involved in osteogenic differentiation, osteogenesis, bone remodeling, and other bone metabolism-related processes [72,73]. To address this topic, our research group investigated the interconnections between transcriptomic profile and epigenetics of the *VDR* gene in a Caucasian cohort consisting of 25 osteoporotic patients (OPs) and 25 healthy subjects (CTRs) [40]. Our analysis identified lower *VDR* expression levels in PBMCs from OPs compared to CTRs, which positively correlates with BMD and *t*-score values, thus associating with a more severe pathological phenotype. *VDR* altered expression levels led us to investigate the *VDR* methylation signature at six CpGs within a 104-bp region located in a *VDR* promoter CGI (CGI 1062; Figure 1) by pyrosequencing analysis. Unfortunately, no statistically significant difference was found in the *VDR* methylation pattern between OPs and CTRs, nor any association between the methylation pattern of the *VDR* and its altered expression levels. These results could be attributable to the presence of additional CGIs/CpG sites not taken into consideration, or to other epigenetic signatures that modulate *VDR* expression levels in OP’s diseases [40]. The expression of the VDR in the male reproductive system and spermatozoa led researchers to investigate the *VDR* methylation signatures in association with reproductive disorders, including male infertility [41,74] (Table 1). These conditions are linked to the supposed positive role of 25(OH)D_3_ in sperm maturation, which would result in increased intracellular calcium concentration, sperm motility, and induction of the acrosome reaction [74]. In this pathological context, Vladoiu and colleagues reported higher *VDR* methylation levels in three promoter CGIs (Island 1- 192 bp, Island 2- 187 bp, Island 3- 160 bp; Figure 1) in infertile males with lower 25(OH)D_3_ levels, compared to males with higher 25(OH)D_3_ levels and healthy subjects [41]. Additionally, the *VDR* methylation percentage increases with the severity of the diagnosis, correlating with lower sperm motility, lower sperm concentration, and altered sperm morphology [41]. These promising results were also confirmed in a subsequent study conducted on a cohort of Egyptian men with idiopathic infertility. Published data reported a negative correlation between the seminal methylation status of CGI2, located in the *VDR* promoter region, and both sperm concentration and progressive motility, strengthening the potential role of this epigenetic mechanism in male infertility [42] (Figure 2). The methylation of the *VDR* has also recently been investigated in the pathological state of recurrent kidney stone formation, given the significant association between the genetic variability of *VDR* and urinary stone formation risks [43,75] (Table 1). In the study conducted by Khatami et al. *VDR* methylation analysis was performed in 30 consecutive recurrent kidney stone formers and 30 age and gender-matched CTRs, revealing a hypermethylation pattern of two *VDR* promoter regions, including 5 and 11 CpG sites, in cases related to CTRs [43] (Figure 2). However, these preliminary analyses need to be further explored as they represent the only bibliographic data available to date on the role of *VDR* methylation in recurrent stone formation. In the context of T2DM, *VDR* methylation has also been analyzed in association with physical activity in a 1:1 matching case–control study [44]. The HRM approach was performed to determine the methylation levels of the *VDR* promoter in a 300 bp region comprissing 27 CpGs, both in 272 T2DM patients and healthy CTRs. Interestingly, the proactive physical activity seems to reduce the risk of T2DM, especially in people with low *VDR* methylation levels. These results suggest that *VDR* methylation could attenuate the association between physical activity and T2DM. Moreover, the increased methylation levels of the *VDR* were also associated with decreased levels of serum insulin, confirming its pivotal role in modulating T2DM phenotype [44] (Figure 2). All these preliminary data encourage additional research on the role of *VDR* methylation in the above-mentioned diseases, which is still poorly explored. To date, the major limitation has been the small region of the *VDR* gene analyzed, which comprise a few CGIs/CpG sites located mainly in the promoter, thus neglecting the entire gene that may have other methylation-sensitive sequences. In addition, further studies on larger sample sizes are needed to confirm the methylation pattern of the *VDR*, as well as functional studies to confirm the role of DNA methylation in these human disorders. 

## 7. New Advances in Therapeutic Strategies

**Figure 3:**
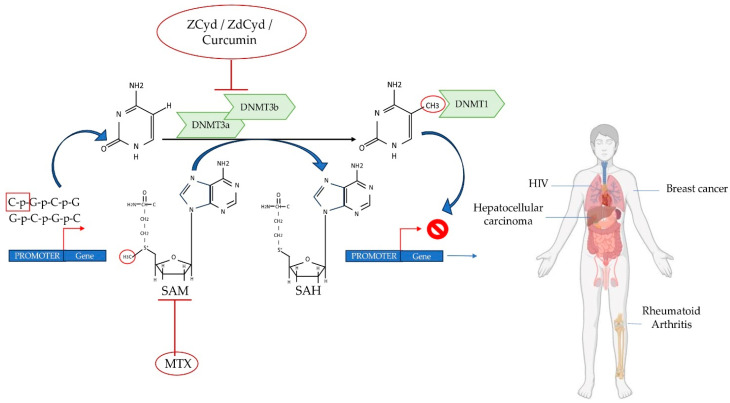
Summary of the potential mechanisms of action of DNA methylation inhibitors in RA, HIV, and cancer. The transfer of a methyl group to the C5 position of cytosine at CpG sites, facilitated by enzymes belonging to DNMTs, results in the formation of 5-methylcytosine. DNMT3a/3b can extract CH3 from the methyl donor SAM to produce SAH. MTX, utilized as an anti-inflammatory drug, has the ability to inhibit SAM. Demethylating agents such as ZCyd, ZdCyd and curcumin can inhibit DNMT3a and DNMT3b activities. DNA methyltransferases, DNMTs; SAM, S-adenosylmethionine; SAH, S-adenosylhomocysteine; ZCyd, 5-Azacytidine; ZdCyd, 5-Aza-2′-deoxycytidine MTX, created with the support of BioRender.com.

Methylation signatures are increasingly being studied as part of new pharmacological strategies due to their ability to be potentially reversible. Several demethylating agents capable of removing DNA methylation aberrations have been identified in the pathological context of autoimmune diseases, infectious diseases, and cancer [76,77]. It has been demonstrated in vitro and in vivo that Methotrexate (MTX), a drug commonly used to treat various diseases, including RA, is able to modulate DNA methylation (Figure 3) [78,79]. MTX inhibits methionine S-adenosyltransferase (MAT), the enzyme responsible for the synthesis of S-adenosyl methionine (SAM), which is able to donate its methyl group in numerous biologically important reactions, including DNA methylation [79]. To date, only one study has explored the role of MTX as an agent capable of modulating the methylation pattern of the *VDR* for the treatment of RA, but, unfortunately, without any positive results [26]. 5-Aza-2′-deoxycytidine (ZdCyd) is a second demethylating agent that has been tested in the field of infectious diseases [34]. An in vitro study performed on TCs previously activated with HIV showed an upregulation of *DNMT3b*, an increased methylation of the *VDR* promoter, and a downregulation of *VDR* levels. Treatment with ZdCyd inhibits the effect of HIV, reverting *VDR* expression patterns [34]. Another class of demethylating agents are chemotherapeutic compounds, normally used as a therapeutic strategy in the treatment of cancer, which are also capable of establishing covalent complexes with DNMTs, leading to the depletion of active enzymes [80]. Among them, 5-azacytidine (ZCyd) was investigated as a treatment agent in calcitriol-resistant breast cancer cell lines. Interestingly, ZCyd induces the hypermethylation of the *VDR* promoter with the consequent decreased *VDR* expression levels. Therefore, ZCyd was able to inhibit the epigenetic silencing of *VDR* and restore calcitriol sensitivity of breast cancer cell lines, thus representing a potential drug in anti-cancer therapy [35]. In the field of HCC, an interesting potential therapeutic molecule is represented by the curcumin as an epigenetic agent. Curcumin is able to covalently block the catalytic site of DNMTs, thus generating an inhibitory effect on DNA methylation signature [81]. In curcumin-treated HepG2 cells, the *VDR* methylation percentage is decreased, and the *VDR* expression pattern is reactivated [37]. All these therapeutic strategies proposed to inhibit aberrant *VDR* methylation represent interesting interventions, which, unfortunately, have several disadvantages. These compounds show cytotoxicity at high doses or long-term treatment [80]. Their active form has a short half-life in circulation and is unstable in aqueous solution [82]. Sometimes, demethylating agents lack specificity for target genes, and this may cause undesirable effects. Finally, some of these compounds, such as ZCyd and ZdCyd, require actively proliferating cells to interact with the DNA [82]. Further studies on the role of *VDR* methylation in various human disorders and the mechanism of action of demethylating agents are essential to translate from in vitro and in vivo studies into clinical practice.

## 8. Conclusions

The *VDR* epigenetic signature through the DNA methylation mechanism emerges as a critical factor in the susceptibility to several human diseases. Currently, there are many gaps and conflicting data on the role of *VDR* methylation in autoimmune diseases, mainly attributable to the lack of a complete understanding of their pathogenic mechanisms. Nevertheless, many studies on infectious diseases and cancer agree that the hypermethylation of the *VDR* promoter is associated with reduced *VDR* expression levels. This signature appears to increase susceptibility to infectious diseases and inhibit the antitumor effects of 1,25(OH)_2_D_3_, thereby increasing cancer risk and progression. However, the use of different detection methods to investigate *VDR* methylation, together with the small sample size and stratification, still has major limitations to be overcome. Furthermore, only few studies directly correlate the *VDR* methylation studies with its expression profile. In this context, the use of bioinformatic databases providing a general overview of gene expression, such as the Genotype-Tissue Expression (GTEx) project, may be useful to integrate epigenetic and expression data [83]. The key role of the VDR in all these pathological conditions underlines the pleiotropy of this factor, prompting researchers to deeply characterize the *VDR* methylation signature and to develop new therapeutic strategies. So far, therapeutic strategies proposed to inhibit aberrant *VDR* methylation have primarily been tested using in vivo and in vitro models, which need to be strengthened to translate preclinical efficacy into practice, improving clinical management and responses to 1,25(OH)_2_D_3_ in human disorders.

## Figures and Tables

**Table 1:** Summary of the main studies on *VDR* gene methylation in autoimmune and infectious diseases, cancer, and other pathological conditions discussed in the text.

	Disease	Sample Size	Method of Analysis	Genomic Context (*VDR* Gene)	Analysis Outcome	Reference
Autoimmune diseases	Rheumatoid arthritis	122 RA vs. 123 CTRs	MethylTarget	CpG sites in the promoter region	*VDR* methylation levels in RA patients were significantly reduced compared to CTRs	[26]
		35 RA vs. 41 CTRs	Pyrosequencing	10 CpG sites in the promoter region	Methylation analysis revealed no significant differences between RA patients compared to CTRs	[22]
	Multiple sclerosis	23 RRMS vs. 12 CTRs	Bisulfite cloning sequencing	23 CpG sites in main promoter, 10 CpG sites in alternative promoter located at non-coding exon 1c	Methylation levels in *VDR* alternative promoter were significantly higher in RRMS patients compared to CTRs	[23]
	Behcet’s disease	48 BD vs. 60 CTRs	MeDIP-qPCR	All CpG sites in the promoter region from −800 bp to +200 bp relative to the TSS	Methylation analysis revealed no significant differences between BD patients compared to CTRs	[30]
Infectious diseases	Tuberculosis disease	27 TB vs. 30 CTRs	Bisulfite cloning sequencing	16 CpG sites in *VDR*	TB patients were in the hypermethylation state compared to CTRs	[31]
		43 TB vs. 33 CTRs	MS-PCR	The location of CpG sites and CGIs present in the *VDR* sequence were identified by DBCAT	Methylation analysis revealed a hypermethylation in TB patients and hypomethylation in CTRs	[13]
		122 TB vs. 118 CTRs	Illumina MiSeq	60 CpG sites in the promoter region (48,299,590–48,298,885)	Methylation levels were significantly lower in TB patients compared to CTRs	[32]
	Hand, foot, and mouth disease	116 HFMD vs. 60 CTRs	MethylTarget	12 CpGs in promoter region from −638 bp to −545 bp relative to the TSS	Methylation levels of *VDR* promoter in HFMD were lower compared to CTRs	[33]
	HIV	TCs obtained from healthy volunteers	Pyrosequencing	CpG in *VDR* promoter region from −512 bp to −28 bp relative to the TSS	HIV-infected TCs showed increased methylation in CpG sites	[34]
Cancer	Breast cancer	15 BCT vs. 7 NBT	MS-PCR	3 CGIs in *VDR* promoter region from −789 bp to +380 bp relative to the TSS	Methylation levels of *VDR* promoter in BCT were significantly higher compared to NBT	[35]
	Adrenocortical carcinoma	23 AT vs. 3 NAT	BSP	42 CpG sites in *VDR* promoter region from −693 bp to −65 bp relative to the TSS	27/42 CpG sites were methylated in 3 ACCs	[36]
	Hepatocellular carcinoma	15 HC vs. 15 CLD vs. 15 NT	MS-PCR	*VDR* promoter	Methylation levels of *VDR* promoter in HCC were significantly higher compared to other studies groups	[37]
	Parathyroid adenomas	15 PAT vs. 4 NPT	BSP	31 CpG sites in *VDR* promoter region from −538 bp to −79 bp relative to the TSS	There was no significant methylation in the promoter region of *VDR* in parathyroid adenomatous tissues	[38]
	Colorectal cancer	75 CCT vs. 75 NE	MS-PCR	*VDR* promoter	Hypermethylation of *VDR* was detected in 28 (37,33%) of 75 cases	[39]
Others	Osteoporosis	25 OP vs. 25 CTRs	Pyrosequencing	6 CpG sites in *VDR* promoter	No statistically significant difference was found in the methylation pattern between OP and CTRs	[40]
	Male infertility	69 ID vs. 37 CTRs	MS-PCR	3 CGIs in *VDR* promoter	*VDR* methylation percentage was increased with the severity of the diagnosis, correlating with lower sperm motility and concentration, and altered sperm morphology	[41]
		60 IID vs. 60 CTRs	MS-PCR	1 CGI in *VDR* promoter	Methylation levels of *VDR* promoter in IID were significantly higher compared to CTRs	[42]
	Recurrent kidney stone formation	30 consecutive recurrent kidney stone formers vs. 30 CTRs	MS-HRM	16 CpG sites in *VDR* promoter	Two *VDR* promoter regions were hypermethylated in patients with consecutive recurrent kidney stone formers compared to CTRs	[43]
	Type 2 diabetes mellitus	272 T2DM vs. 272 CTRs	MS-HRM	27 CpG sites in *VDR* promoter	Increased methylation levels of *VDR* were associated with decreased levels of serum insulin	[44]

Abbreviations: RA, rheumatoid arthritis; CTRs, control groups; VDR, vitamin D receptor; RRMS, relapsing-remitting multiple sclerosis; BD, Behcet’s disease; MeDIP-qPCR, methylated DNA immunoprecipitation-real time PCR; TSS, transcription start site; TB, tuberculosis; MS-PCR, methylation specific-polymerase chain reaction; CGIs, CpG islands; DBCAT, DataBase of CGIs and analytical tool; HFMD, hand, foot, and mouth disease; HIV, human immunodeficiency virus; TCs, Tcell; BCT, breast cancer tissue; NBT, normal breast tissue; AT, adrenal tumors; NAT, normal adrenal tissue; BSP, bisulfite sequencing PCR; ACC, adrenocortical carcinoma; HC, hepatocellular carcinoma; CLD, chronic liver disease; NT, normal tissue; PAT, parathyroid adenomas tissue; NPT, normal parathyroid tissue; CCT, colorectal cancer tissue; NE, normal epithelium; OP, osteoporosis; ID, infertility disease; IID, idiopathic infertility disease; MS-HRM, methylation-sensitive high-resolution melting; T2DM, type 2 diabetes mellitus.

## Data Availability

The data presented in this study were extracted from the articles cited in the text.
